# Selenium deficiency is functionally linked with the molecular etiopathogenesis of necrotizing enterocolitis (NEC)

**DOI:** 10.1007/s10142-025-01628-8

**Published:** 2025-06-03

**Authors:** Kubilay Gürünlüoğlu, Muhammed Dündar, Turgay Ünver, Hatice Turgut, Semra Gürünlüoğlu, Necmettin Akpınar, Hasan Ateş, Ramazan Özdemir, Turan Yıldız, Mehmet Demircan, Mehmet Aslan, Ahmet Koç

**Affiliations:** 1 Department of Pediatric Surgery, Faculty of Medicine, Inonu University, Malatya, Türkiye; 2 Department of Medical Genetics, Faculty of Medicine, Inonu University, Malatya, Türkiye; 3 Ficus Biotechnology, Ostim, Yenimahalle, Ankara Türkiye; 4 Department of Pediatrics and Division of Neonatology, Faculty of Medicine, Inonu University, Malatya, Türkiye; 5 Department of Pathology, Faculty of Medicine, Inonu University, Malatya, Türkiye; 6 Department of Pediatrics and Division of Emergency, Faculty of Medicine, Inonu University, Malatya, Türkiye

**Keywords:** Hypoxia-induced apoptosis, Necrotizing Enterocolitis, Premature infants, Selenium deficiency

## Abstract

Necrotizing enterocolitis (NEC) is a severe and often catastrophic gastrointestinal emergency that predominantly affects neonates, especially those born prematurely, and is associated with high rates of morbidity and mortality. Despite its significant clinical impact, the precise etiology and molecular pathogenesis of NEC remain incompletely understood. In this study, we conducted global transcriptomic profiling using high-throughput RNA sequencing in 11 premature neonates diagnosed with NEC, following rigorous inclusion and exclusion criteria. Compared to healthy controls, we identified 1,204 differentially expressed genes (DEGs), including 636 upregulated and 568 downregulated transcripts. Notably, genes involved in hypoxia-induced apoptosis (e.g., *HIF1 AAS3*, *HIF1 AAS1*), the caspase cascade (*BCL2*, *BCL6*, *CASP5*, *CASP7*), and inflammation (*IL1RAP*, *IL6ST*, *TNFAIP3*, *TNFRSF10 A*, *TLR6*, *TLR10*) were significantly upregulated. In contrast, *IL18*, a key modulator of inflammatory responses, was downregulated. Interestingly, several genes encoding selenoproteins (*GPX1*, *GPX4*, *SELENON*, *SELENOM*, *SELENOF*, *SELENOW*, *SELENOT*) were also downregulated, suggesting molecular evidence of selenium deficiency. Gene ontology and pathway enrichment analyses revealed widespread dysregulation in pathways related to hypoxia response, systemic inflammation, coagulation, antimicrobial defense, mitochondrial function, autophagy, selenium metabolism, and apoptosis. Collectively, our findings provide novel insights into the molecular underpinnings of NEC in premature infants and suggest that systemic hypoxia, oxidative stress, selenium deficiency, and programmed cell death contribute significantly to its pathogenesis.

**Supplementary Information:**

The online version contains supplementary material available at 10.1007/s10142-025-01628-8.

## Introduction

Necrotizing enterocolitis (NEC), whose etiology is not fully understood, is a common neonatal disease that particularly affects preterm infants, requires gastrointestinal emergency surgical intervention, represents a leading cause of mortality and morbidity in neonatal intensive care units worldwide, and is characterized by severe thrombocytopenia, widespread intravascular coagulopathy and intestinal ischemia. Although its etiology remains unclear, prematurity, postprandial mucosal compromise, and inflammation following bowel injury are believed to play key roles in the pathogenesis of NEC (Kim and Albenese 2006).

The following common clinical scenario supports a unified hypothesis for the pathogenesis of NEC. In premature infants, respiratory distress arises due to underdeveloped respiratory systems. As a result of this immaturity, episodes of apnea and bradycardia are common, leading to reduced blood perfusion in the intestinal mucosa. The ensuing hypoperfusion triggers a sudden increase in apoptosis within the intestinal epithelium, compromising the mucosal barrier and initiating the release of proinflammatory cytokines and activation of the inflammatory cascade. This cascade further increases mucosal permeability, exacerbating injury to both the mucosa and the bowel wall. Consequently, bacteria and their by-products translocate across the damaged mucosa, leading to progressive bowel injury, full-thickness necrosis, and eventual perforation (Mannoia et al. 2011).

We propose that the development of NEC results from a cascade of interrelated events that, while seemingly independent, ultimately converge to initiate the disease in a manner analogous to a domino effect. Key contributing factors in this sequence include selenium deficiency, prematurity, systemic oxidative stress, widespread inflammation, and increased apoptosis. In this context, we aim to underscore the significance of each of these factors and their collective role in the pathogenesis of NEC.

A strong correlation exists between prematurity and the incidence of NEC. NEC affects approximately 7,2% of newborns and remains one of the leading causes of neonatal mortality (Llanos et al. 2002). While the typical gestational period is 40 weeks, infants born before 37 weeks are classified as preterm, and those born before 28 weeks represent the highest risk group for NEC (Garg et al. 2023). Gastrointestinal tract maturation is largely completed by 35 weeks of gestation. The precise causes of preterm birth remain unclear, particularly in pregnancies without any identifiable fetal anomalies. However, some studies have suggested a potential association between maternal selenium deficiency and premature delivery (Gathwala et al. 2003; Gathwala and Aggarwal 2016). In a recent study investigating gene expression profiles in premature infants, significant alterations were observed in the expression of *KLRB1*, *KLRD1*, *CREBBP*, and *HIF1 A* genes (Hilgendorff et al. 2017), suggesting a possible link between molecular dysregulation and the pathophysiological mechanisms underlying NEC.

Oxidative stress is recognized as one of the contributing factors to preterm birth during pregnancy (Phillippe 2022). Additionally, oxidative stress in premature infants may play a pivotal role in the pathogenesis of NEC (Aceti et al. 2018). In healthy individuals, cellular metabolic processes generate various toxic by-products on a daily basis, including reactive oxygen species (ROS), which are harmful to cells and tissues (Aydemir et al. 2011; Marseglia et al. 2014). The cumulative burden of these oxidative substances that the body must neutralize is referred to as the total oxidant capacity (TOC) (Aydemir et al. 2011; Marseglia et al. 2014). Conversely, the sum of all antioxidant elements, enzymes, and compounds that the body utilizes to counteract the effects of oxidants and protect against cellular damage is termed total antioxidant capacity (TAC) (Aydemir et al. 2011; Marseglia et al. 2014). Oxidative stress arises when this balance is disrupted in favor of TOC, due to either an increase in oxidant production or a reduction in antioxidant defenses. Such an imbalance commonly occurs in conditions characterized by extensive cellular injury, hypoxia, or severe trauma, such as burns (Aydemir et al. 2011; Marseglia et al. 2014).

Systemic oxidative stress not only initiates systemic inflammation through a cascade of biological events but also promotes increased apoptosis across various cell types due to the detrimental effects of this inflammation (Marseglia et al. 2014). To date, several studies have reported altered expression of genes such as *HIF1 AAS1*, *TLR6*, *GPX1*, *GPX4*, *SELENOM*, *IL1R2*, *IL6ST*, *IL6RA*, *IL18*, *TNFAIP3*, and *TNFRSF10 A* in response to hypoxia, oxidative stress, and elevated inflammatory states (Semenza 2001; Wu et al. 2023; Cecrdlova et al. 2016). Programmed cell death occurring under physiological conditions is referred to as apoptosis, which can be triggered by factors such as systemic oxidative stress and inflammation (Marseglia et al. 2014; Semenza 2001; Wu et al. 2023; Cecrdlova et al. 2016; Nagata 2018). In the unified hypothesis widely accepted for NEC pathogenesis, a sudden and unexplained increase in apoptosis represents a critical turning point in the cascade of events leading to the disease (Kim and Albenese 2006). This apoptotic surge, particularly in the intestinal villus crypts, is believed to compromise the integrity of the mucosal barrier (Kim and Albenese 2006). Recent studies have reported altered expression of genes such as *ACTB*, *HIF1 AAS3*, *HIF1 AAS1*, *CASP5*, *BCL2*, *BCL6*, and *CASP7* in association with increased apoptosis in intestinal villus crypts (Kwon et al. 2013; Bai et al. 2020).

Selenium is an essential trace element that is incorporated into the structure of selenoproteins and plays crucial roles in several metabolic pathways (Lammi and Qu 2018). Selenoproteins synthesized from selenium are vital for maintaining proper immune system function (Reeves and Hoffmann 2009; Huang et al. 2012; Salaramoli et al. 2022). In addition to initiating immune responses, selenoproteins are essential for regulating excessive immune activation and preventing chronic inflammation (Huang et al. 2012). At the molecular level, they contribute to immune cell differentiation—particularly T cell development in the spleen—through intracellular signaling pathways (Huang et al. 2012). Moreover, selenoproteins protect cells from oxidative stress by regulating intracellular calcium influx, scavenging reactive oxygen species (ROS), and mitigating oxidative bursts and lipid peroxidation-induced damage (Salaramoli et al. 2022). These protective mechanisms allow selenoproteins to effectively regulate systemic oxidative stress and inflammation, thereby enhancing cellular resistance to oxidative injury (Salaramoli et al. 2022). Importantly, these actions also help prevent apoptosis via the mitochondrial pathway, highlighting the pivotal role of selenoproteins in maintaining cellular homeostasis and immune defense (Reeves and Hoffmann 2009; Huang et al. 2012; Salaramoli et al. 2022). It has been suggested that selenium deficiency in pregnant women is associated with an increased risk of preterm birth (Gathwala and Aggarwal 2016). Since selenium is predominantly transferred to the fetus during the third trimester, preterm infants are particularly vulnerable to selenium deficiency (Tindell and Tipple 2018). Genes involved in selenium uptake and transport, which may be dysregulated in states of selenium deficiency, include *SELENOID*, *GPX1*, *GPX4*, *SELENON*, *SELENIUM*, *SELENOF*, *SELENOW*, *SELENOT*, *SELENO*, and *SEPHS2* (Santesmasses et al. 2020; Hesketh 2008).

To date, a limited number of transcriptome analyses performed on premature infants with NEC (Jung et al. 2017; Egozi et al. 2023; Xie et al. 2023). In these studies, NEC was not considered a systemic disease, but was evaluated as a local bowel disease in which some intestinal segments were affected. Therefore, the gene expression pattern was measured in the affected intestinal segments of NEC patients (Jung et al. 2017; Egozi et al. 2023; Xie et al. 2023). In contrast, the present study aims to explore the global gene expression landscape in NEC patients and compare it with that of healthy controls using high-throughput RNA sequencing technology.

## Materials and methods

### NEC Patients and sampling

**Fig. 1:**
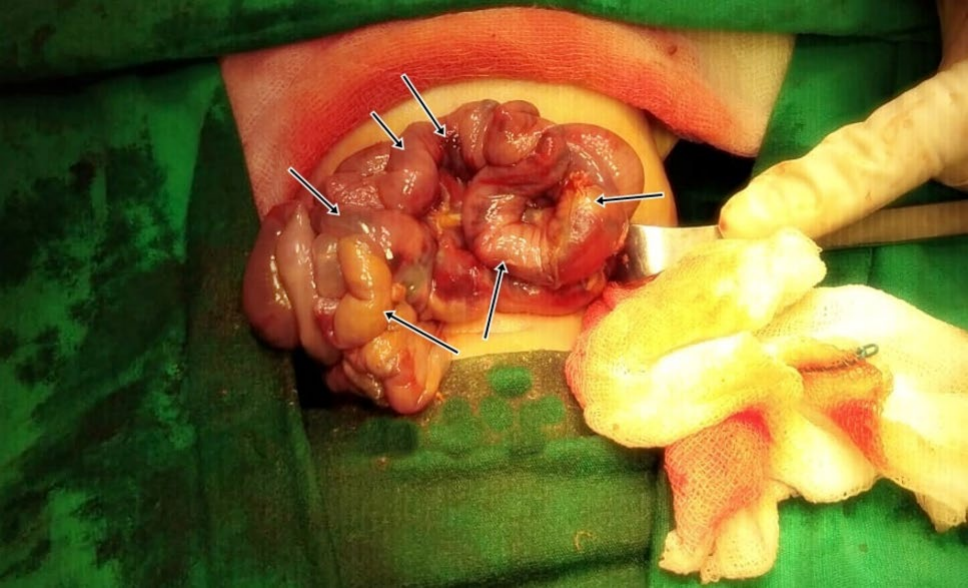
Intraoperative view of the intestines of a premature infant operated for NEC. The arrows indicate intestinal areas with impaired blood circulation of different severity

This study was designed as a single-center investigation. All patients, including the control infant, were selected from the Pediatric Neonatal Intensive Care Unit of İnönü University Turgut Özal Medical Center. The study was conducted in accordance with the principles of the Declaration of Helsinki, and informed consent was obtained from the parents of all participating infants. During the design phase of this study, we initially intended to include a larger control group; however, this was not permitted by the ethics committee. We recognize that multiple factors associated with prematurity, such as immature respiratory function and hypoxia, contribute to the complex pathogenesis of NEC and may act as critical components of its development. To ensure an appropriate comparison group without confounding variables, we selected control infant who was healthy and exhibited no such complications. The study was conducted over a period spanning from July 2022 to November 2024**.** The study included 11 premature infants diagnosed with NEC and one healthy control infant, all selected based on strict inclusion and exclusion criteria. The inclusion criteria for NEC patients were: being born prematurely (gestational age < 37 weeks), having a confirmed diagnosis of Bell Stage 3 NEC, undergoing emergency surgery due to NEC (Fig. 1), and the availability of a preoperative blood sample for genetic analysis. These criteria were established to ensure a homogeneous study population and to facilitate reliable interpretation of gene expression data in the context of severe NEC. The exclusion criteria for NEC patients included: receiving blood or blood products, being classified as Bell Stage 1 or 2, the presence of any major congenital anomaly, being born at term (≥ 37 weeks of gestation), and not undergoing surgical intervention for NEC. The control infant in the study was a full-term neonate with normal birth weight, no medical conditions, and no history of treatment. The Bell staging system, which defines the diagnostic criteria for NEC, is presented in Supplementary Table 1. Demographic information for all infants included in the study is provided in Supplementary Table 2.

Direct abdominal X-rays revealed pneumoperitoneum in all patients. Based on the Bell staging system, all patients were diagnosed with Stage 3 NEC and underwent emergency surgical intervention. Blood samples were collected from each patient as previously described (Gürünlüoğlu et al. 2022). Briefly, 2–3 mL of intravenous blood was drawn from the right or left forearm using the Seldinger technique.

### RNA Isolation

Total RNA was isolated directly from whole blood samples of both patient and control groups using the QIAamp RNA Blood Mini Kit (Qiagen, Hilden, Germany) following the manufacturer’s instructions. Subsequently, RNA concentration was determined with a spectrophotometer (Denovix DS-11 FX +, Wilmington, USA) and preserved at −80 °C.

### Library preparation and sequencing

RNA quantity and integrity were checked with Agilent 5400 system (Agilent, USA). RNA sequencing libraries were generated using NEBNext Ultra RNA Library Prep Kit for Illumina (NEB, USA, Catalog #: E7530L) following manufacturer’s instructions. The index codes were added to adjacent sequences to each sample. In brief, mRNAs were purified from blood total RNAs using poly-T attached magnetic beads.Tagmentations were performed via divalent cations under elevated temperature in NEB Next First Strand Synthesis Reaction Buffer (5X). Then the first strand cDNAs were synthesized usingrandom hexamer primer and M-MuLV Reverse Transcriptase (RNase H). After that, the second strand cDNAs were synthesized using DNA Polymerase I and RNase H. Remaining overhangs were converted into blunt ends via exonuclease/polymerase and NEB Next Adaptor with hairpin loop structure were ligated to prepare for hybridization. cDNA fragments of preferentially 370 ~ 420 bp in length were selected and purified with AMPure XP system (Beverly, USA). Then libraries were amplified with a High-Fidelity DNA polymerase which were purified (AMPure XP system) and subjected to libraryquality assessment on the Agilent 5400 system (Agilent, USA) and quantified by qPCR as of 1.5 nM in concentration. Finally, the sequencing was performed with the Illumina NovaSeq X platform in a paired end (PE) 2 × 150 bp layout.

### Bioinformatics analysis

Clean reads were aligned to the reference genome (Homo sapiens, assembly GRCh38.p13) using the STAR aligner (Dobin et al. 2013). Low-quality readings with contaminant arrays and adapter were removed using the FASTQC tool < www.bioinformatics.babraham.ac.uk/projects/fastqc/ >. DEGs were measured using the edgeR R package (Robinson et al. 2010). DEGs were selected as following criteria; up regulated log_2_fold change (log_2_FC) > 1 and down-regulated log_2_FC < 1 at a False Discovery Rate (FDR) cut-off 5% (FDR < 0.05, p = 0.05) < http://bioconductor.org/packages/release/bioc/html/edgeR.html >. Quantification of the mapped reads was done using the HTSeq Python library < https://htseq.readthedocs.io/en/master/ > (Anders et al. 2015). Gene ontology (GO) analysis was performed to obtain GO terms (Ashburner et al. 2000) then gene set enrichment with the multiple test correction method of Benjamini-Hochberg (FDR 5%, adjusted p-value of 0.05) to overrepresented GO terms were determined by applying Fisher's Exact Test (Al-Shahrour et al. 2004), and KEGG pathway analysis was conducted (Kanehisa and Goto 2000) in the OmicsBox tool v.3.4.5 < https://www.biobam.com/omicsbox/ >.

## Results

### Patients and control characteristics

All patients diagnosed with necrotizing enterocolitis (NEC) in this study were premature and of low birth weight (Supplementary Table 2). According to the Bell staging system, all cases were classified as Stage 3. The cohort included five male and six female infants (Supplementary Table 2). From the beginning of the study to the time of manuscript preparation, two patients died due to sepsis and respiratory complications, while nine patients remained alive (Supplementary Table 2). The control subject used for comparison was a healthy full-term female infant.

### Differentially expressed genes (DEGs)

High-throughput sequencing data for eleven NEC patients were generated using the Illumina platform. A total of 512 million (M) raw reads were produced, with an average of 23 M paired-end (PE) 150 base pair (bp) × 2 reads per library. The sequencing quality was high, with Q20 scores exceeding 98% and Q30 scores exceeding 95% (Table 1; see also Supplementary Table 3 for additional statistics). After adapter trimming and removal of low-quality reads, approximately 500 M clean reads were obtained (Supplementary Table 3). The cleaned datasets were submitted to the NCBI SRA repository under Submission ID: 15,264,887.

**Table 1:** The experimental design and sequencing statistics of samples

Sample Name	Experimental Design	Platform/ Layout	Raw Reads (Read 1 + Read 2)	Q20 (%)
Control	Control group	Illumina Novaseq, Paired-end (PE)	37,449,888	98.58
Patient 1	Test group	Illumina Novaseq, Paired-end (PE)	49,824,192	98.54
Patient 2	Test group	Illumina Novaseq, Paired-end (PE)	51,970,064	98.56
Patient 3	Test group	Illumina Novaseq, Paired-end (PE)	49,629,482	98.52
Patient 4	Test group	Illumina Novaseq, Paired-end (PE)	43,982,176	98.58
Patient 5	Test group	Illumina Novaseq, Paired-end (PE)	46,328,822	98.53
Patient 6	Test group	Illumina Novaseq, Paired-end (PE)	48,109,192	98.58
Patient 7	Test group	Illumina Novaseq, Paired-end (PE)	39,469,194	98.45
Patient 8	Test group	Illumina Novaseq, Paired-end (PE)	43,084,738	98.43
Patient 9	Test group	Illumina Novaseq, Paired-end (PE)	40,043,064	98.74
Patient 10	Test group	Illumina Novaseq, Paired-end (PE)	45,947,742	98.72
Patient 11	Test group	Illumina Novaseq, Paired-end (PE)	53,570,550	98.45

**Fig. 2:**
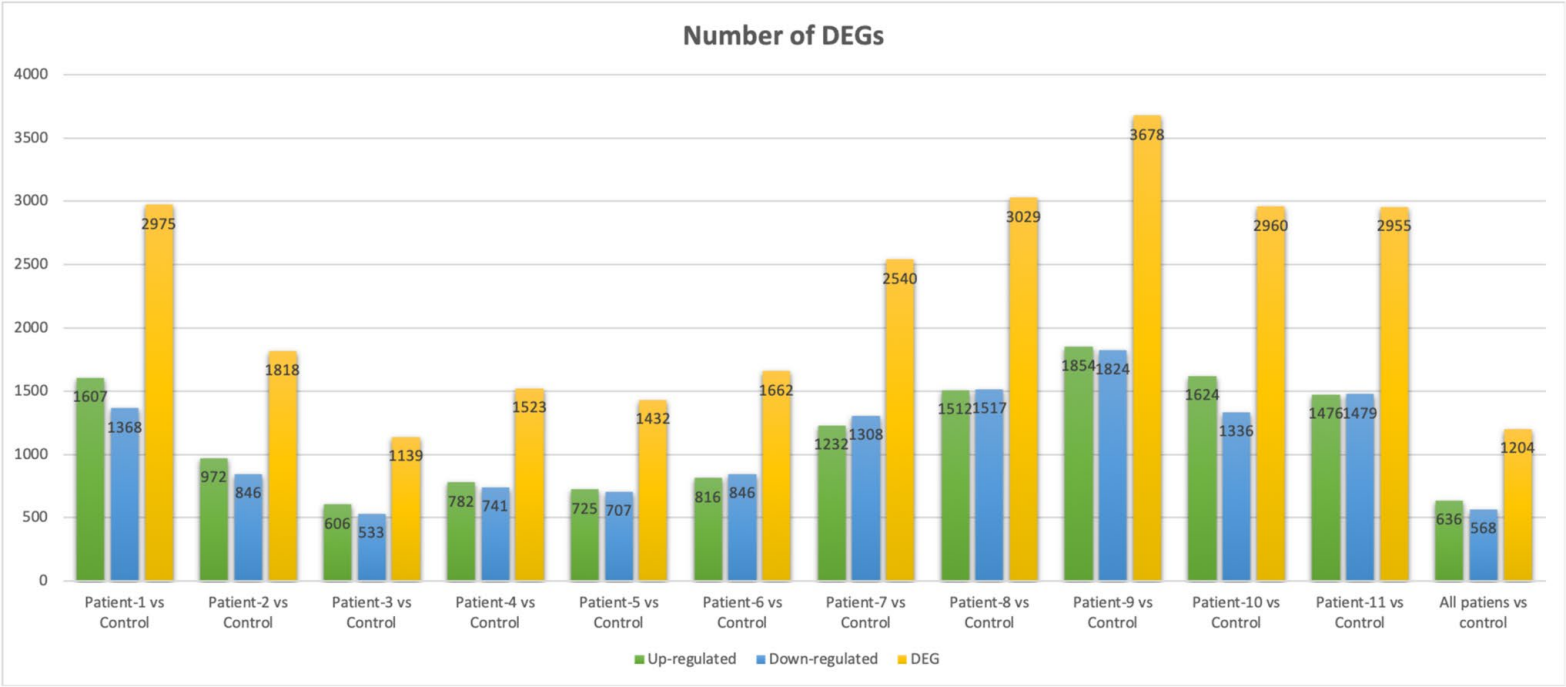
Number of DEGs. A) Up-regulated and down-regulated gene numbers found in comparison with patient versus control transcriptomes. B) Shared DEGs across different patient samples compared to the control

More than 99% of the reads successfully mapped to the human reference genome (hg38; Genome Assembly GRCh38, NCBI) (Table 2). Gene expression levels for each sample were quantified and normalized as counts per million (CPM). Pairwise differential expression analysis, using all NEC patients as the treatment group and the healthy infant as the control, identified a total of 1,204 differentially expressed genes (DEGs), of which 636 were upregulated and 568 were downregulated, based on a log2 fold change (log2 FC) threshold of > 1 or < − 1 (Fig. 2a; Supplementary Tables 4 and 5).

**Table 2:** Reference genome mapping statistics

Sample Name	Mapped Reads (Count)	Mapped Reads (Percentage)	Average Mapped Length (Base)
Control	37,225,247	99.41%	149.66
Patient 1	49,714,616	99.78%	150.00
Patient 2	51,840,759	99.75%	150.00
Patient 3	49,505,609	99.75%	150.00
Patient 4	43,872,879	99.75%	150.00
Patient 5	46,220,212	99.77%	150.00
Patient 6	47,995,124	99.76%	150.00
Patient 7	39,223,005	99.38%	150.00
Patient 8	42,986,400	99.77%	150.00
Patient 9	39,800,858	99.44%	150.00
Patient 10	45,667,182	99.39%	150.00
Patient 11	53,298,329	99.49%	150.00

In individual comparisons of each NEC patient against the control, Patient 9 showed the highest number of DEGs (3,678), while Patient 3 had the lowest (1,139). In all but three cases (Patients 6, 7, and 8), the number of upregulated genes exceeded the number of downregulated genes (Fig. 2a; Supplementary Table 5). Overall, comparative transcriptomic analysis between NEC patients and the control sample revealed 636 consistently upregulated and 568 downregulated genes (Supplementary Table 5). Additionally, shared DEGs across patient samples were identified and visualized (Fig. 2b). The top 20 most significantly upregulated and downregulated genes are presented in Table 3 and Supplementary Fig. 1.

**Table 3:** The DEGs analysis statistics

Comparison Groups	Total DEGs number (Probability > 0.9)	Up-regulated gene number (M > 0)	Down-regulated gene number (M < 0)
patient_1_vs_control	2,975	1,607	1,368
patient_2_vs_control	1,818	972	846
patient_3_vs_control	1,139	606	533
patient_4_vs_control	1,523	782	741
patient_5_vs_control	1,432	725	707
patient_6_vs_control	1,662	816	846
patient_7_vs_control	2,540	1,232	1,308
patient_8_vs_control	3,029	1,512	1,517
patient_9_vs_control	3,678	1,854	1,824
patient_10_vs_control	2,960	1,624	1,336
patient_11_vs_control	2,955	1,467	1,479
**Comparison** **Groups**	**Total** **DEGs number** **(FDR < 0.05)**	**Up-regulated** **gene number** **(logFC > 1)**	**Down-regulated gene number** **(logFC < −1)**
all_patients_vs_control	1,204	636	568

### Functional annotations by gene ontology (GO) and KEGG pathways

**Fig. 3:**
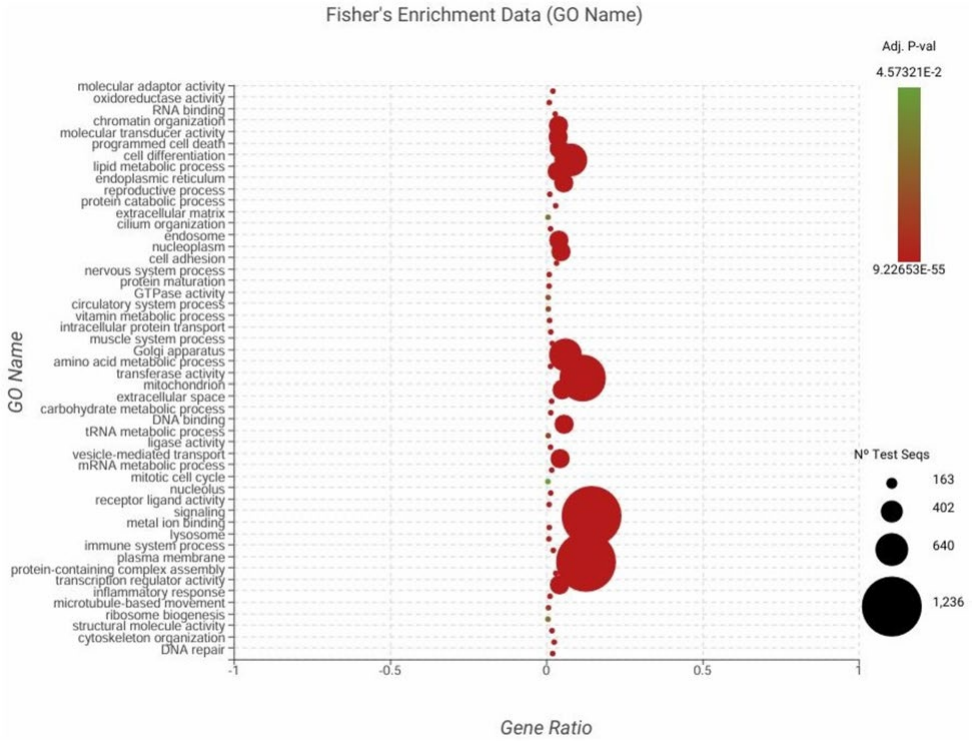
Gene set enrichment analysis of GO terms according to Fisher's Exact Test

**Fig. 4:**
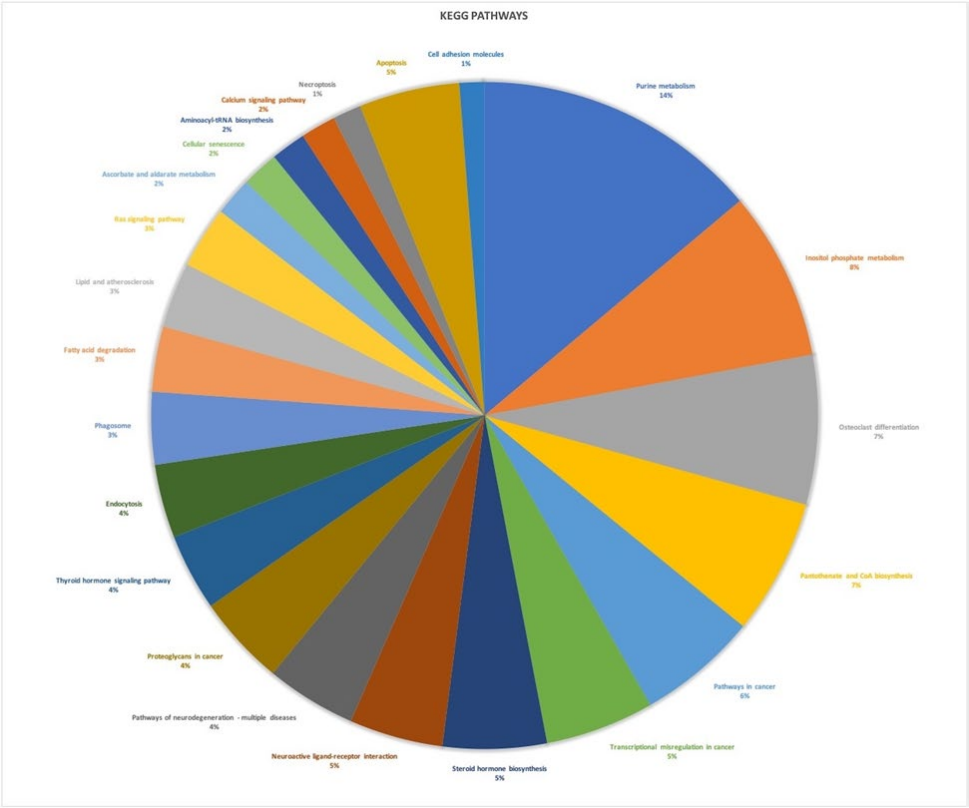
Pie chart representation of KEGG activated molecular stand out pathways

To identify functional roles, the 1,204 differently expressed genes were annotated in terms such as molecular function (MF), cellular component (CC), and biological process (BP) (Supplementary Table 6). Most of the BP terms was identified as cellular process (38.77%) and regulation of biological process (22.41%) (Supplementary Fig. 2). Catalytic activity (53.4%) and binding (36.35%) are the main MF terms (Supplementary Fig. 3). Since thousands of the sequences matching with several GO terms as MF, BP and CC (Supplementary Table 6), a gene set enrichment procedure was applied to determine the set of genes that are statistically significant. A total of 216 enriched GO terms was listed (Supplementary Table 7). Among them, MF terms are mainly associated with"molecular adaptor activity","programmed cell death","oxido-reductase activity","metal ion binding", and"lipid metabolic process". Furthermore, the terms related to biological processes are related to"inflammatory response","immune system process","metabolic process","developmental process","biological adhesion","developmental process"and"growth". The BP terms were identified mostly as"mitotic cell cycle","mitochondria","DNA repair","cell adhesion", and"lysosome"(Fig. 3, Supplementary Table 7). KEGG pathway analysis identified thiamine metabolism with 194 sequence hits. Additionally, purine metabolism (14%), apoptosis (5%), and phagosome (3%) were among the 314 pathways detected (Fig. 4, Supplementary Table 8).

### Regulation of the genes that might be involved in the NEC etiopathogenesis

**Fig. 5:**
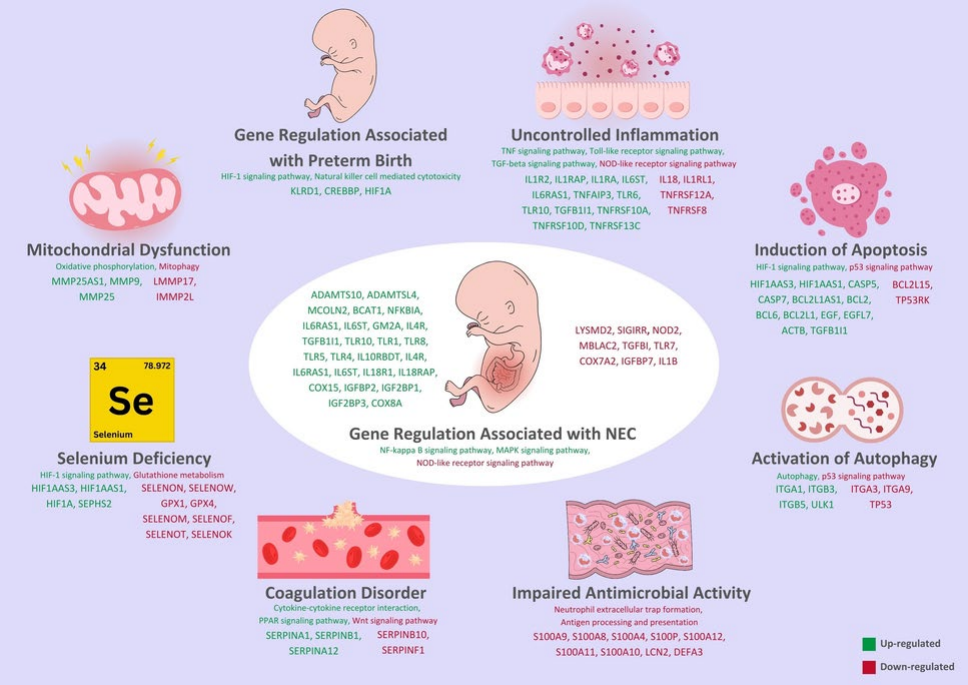
Molecular mechanisms associated with the role of selected differentially expressed genes in the etio-pathogenesis of NEC are shown

Upregulation of genes such as *KLRD1*, *CREBBP*, and *HIF1 A*, which are associated with preterm birth, was observed in NEC patients. In contrast, the expression of inflammation-related genes including *IL18*, *IL1RL1*, *TNFRSF12 A*, and *TNFRSF8* was found to be downregulated in NEC samples. However, several other inflammation-associated genes, including *IL1R2*, *IL1RAP*, *IL1RA*, *IL6ST*, *IL6RA*, *TNFAIP3*, *TLR6*, *TLR10*, *TGFB1I1*, *TNFRSF10 A*, *TNFRSF10D*, and *TNFRSF13 C*, were significantly upregulated in NEC patients (Fig. 5).

Apoptosis-related genes *BCL2L15* and *TP53RK* were consistently downregulated across all patients. In contrast, increased expression was noted in genes involved in apoptotic pathways, including *HIF1 AAS3*, *HIF1 AAS1*, *CASP5*, *CASP7*, *BCL2L1 AS1*, *BCL2*, *BCL6*, *BCL2L1*, *EGF*, *EGFL7*, *ACTB*, and *TGFB1I1*.

Autophagy-related genes *ITGA3*, *ITGA9*, and *TP53* were found to be downregulated in NEC samples, whereas *ITGA1*, *ITGB3*, *ITGB5*, and *ULK1* were upregulated (Fig. 5).

We also identified downregulation of several genes previously reported to play protective roles against NEC, including *LYSMD2*, *SIGIRR*, *NOD2*, *MBLAC2*, *TGFBI*, *TLR7*, *COX7 A2*, *IGFBP7*, and *IL1B*. Conversely, a number of genes implicated in NEC pathogenesis in the literature were significantly upregulated in our dataset, such as *ADAMTS10*, *ADAMTSL4*, *MCOLN2*, *BCAT1*, *NFKBIA*, *IL6RA*, *IL6ST*, *GM2 A*, *IL4R*, *TGFB1I1*, *TLR10*, *TLR1*, *TLR8*, *TLR5*, *TLR4*, *IL10RBDT*, *IL18R1*, *IL18RAP*, *COX15*, *IGFBP2*, *IGF2BP1*, *IGF2BP3*, and *COX8 A* (Fig. 5).

Furthermore, genes associated with selenium uptake and transport, including *SELENON*, *SELENOW*, *GPX1*, *GPX4*, *SELENOM*, *SELENOF*, *SELENOT*, and *SELENOK*, were downregulated. In contrast, genes associated with selenium deficiency such as *HIF1 AAS3*, *HIF1 AAS1*, *HIF1 A*, and *SEPHS2* were upregulated (Fig. 5).

Transcriptome analysis also revealed consistent downregulation of antimicrobial activity genes, including *S100 A9*, *S100 A8*, *S100 A4*, *S100P*, *S100 A12*, *S100 A11*, *S100 A10*, *LCN2*, and *DEFA3* (Fig. 5).

With respect to coagulation regulation, *SERPINB10* and *SERPINF1* were downregulated, while *SERPINB1*, *SERPINA1*, and *SERPINA12* were upregulated (Fig. 5).

In addition, genes related to mitochondrial function were differentially expressed in NEC patients. For instance, *LMMP17* and *IMMP2L* were suppressed, whereas *MMP25 AS1*, *MMP9*, and *MMP25* were upregulated (Fig. 5).

## Discussion

**Fig. 6:**
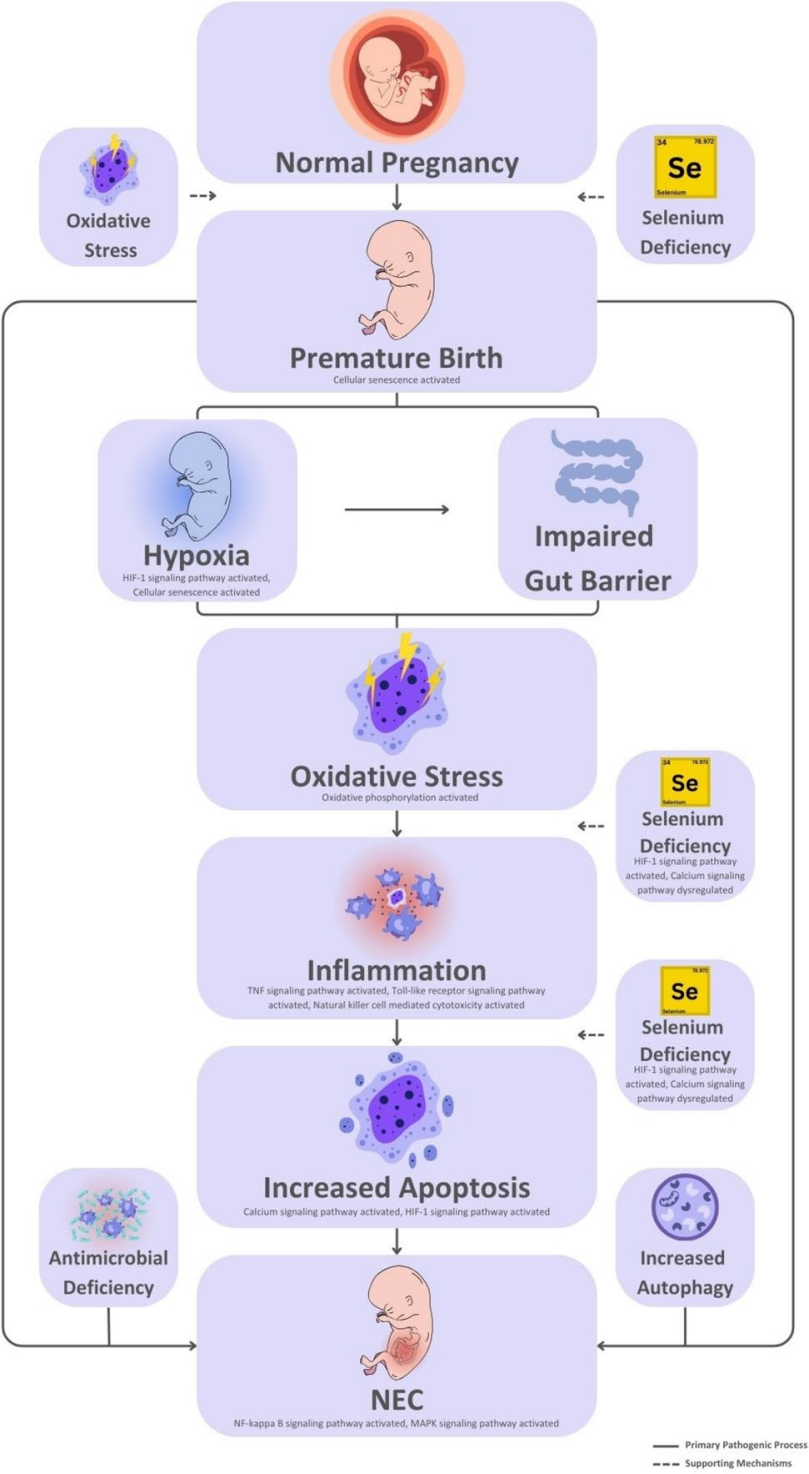
Global gene expression profiling helps to identify the molecular representation of NEC etiopathogenesis

Global gene expression profiling in premature infants with necrotizing enterocolitis (NEC), utilizing comparative RNA sequencing analysis, revealed several notable transcriptomic alterations. Our results showed dysregulation of hypoxia-responsive genes in accordance with hypoxic exposure in these infants. Gene expression of oxidative stress response and inflammatory pathway genes was drastically changed, indicating an enhanced systemic inflammatory condition. Downregulation of antimicrobial defense-related genes could indicate impaired innate immunity. Autophagy-related gene expression was also found to be changed, indicating disruption of cellular homeostasis. Further, expression patterns of the genes revealed a lack of selenium-related pathways, selenium being a crucial element in lessening oxidative stress and inflammation control. Interestingly, perhaps the most salient observation was an upregulation of the apoptosis genes with enhanced programmed cell death in NEC-infants. Based on our findings and current literature, we formulated a hypothesis regarding the pathogenesis of NEC in preterm infants, as indicated in Fig. 6. We propose that maternal oxidative stress, aggravated by increased oxidant exposure and selenium deficiency during pregnancy, may lead to telomere shortening in placental cells, resulting in preterm birth. As a result, preterm infants, whose respiratory tracts are less developed, may be particularly susceptible to hypoxia. Hypoxia can undermine intestinal perfusion and cause mucosal damage. Such early movements can trigger systemic oxidative stress and inflammation in the premature neonate.

Selenium, another critical trace element of the antioxidant defense system, is also frequently lacking in premature infants, further undermining their ability to withstand oxidative damage. We suggest that one of the first and most important events in NEC pathogenesis is increased apoptosis, arising consequence of these stress factors. The cascade of etiopathogenesis is also driven by disruption of antimicrobial defense systems and enhanced autophagy. In general, we think oxidative stress and selenium deficiency are important, interlinked causes, radiating in the mother and infant, initiating the cascade of NEC. It is important to note that actual selenium levels were not measured in the patient cohort. Instead, this study focused on analyzing gene expression changes associated with selenium deficiency. The conclusions presented are therefore hypothesis-generating and based solely on the observed transcriptomic findings.

Preterm birth and associated immaturity are the principal risk factors that put infants at risk for the development of NEC. Prematurity-related immaturity of the respiratory and gastrointestinal systems often necessitates limited feeding strategies and contributes to hypoxia and respiratory complications in affected neonates (González-Rivera et al. 2011; Yee et al. 2012). However, this does not fully account for the delayed onset of NEC observed in some infants born before 26 weeks of gestation. Studies by González-Rivera et al. and Yee et al. indicate that the relationship between gestational age and NEC onset is not strictly linear (González-Rivera et al. 2011; Yee et al. 2012). We propose that the variability in NEC onset may be due to the differing intensities of predisposing factors present in each infant. In our study, four infants were born at less than 26 weeks’ gestation, and the timing of NEC onset in these cases was comparable to that observed in infants with higher gestational ages (Supplementary Table 2). We propose that prevention of premature birth could significantly reduce the incidence of NEC. Phillippe (Phillippe 2022) proposed that telomere length in placental cells is one of the most important factors in deciding the onset of labor. In a healthy pregnancy, telomeres in the placenta are uniform in length, but premature shortening of telomeres may trigger premature birth. Phillippe indicates that one of the key reasons for telomere shortening in placental cells is maternal oxidative stress and that such oxidative stress is commonly linked with deficiency of maternal antioxidant defenses. He also suggested that telomere shortening accelerates cellular senescence and apoptosis in the placenta, causing preterm birth. Supporting this hypothesis, Gathwala et al. (Gathwala et al. 2003) demonstrated that mothers who delivered preterm had lower maternal serum selenium levels—a key component of the antioxidant defense system—compared to mothers who delivered at term. Collectively, all these findings suggest that maternal oxidative stress and selenium deficiency may be critical upstream events in the cascade leading to premature delivery and, by extension, NEC. Gathwala et al. (Gathwala et al. 2003) in their work discovered that the levels of maternal blood selenium were much lower in preterm-delivering women (63.2 ± 2 µg/L) compared to term-delivering women (70.6 ± 1 µg/L). For reference, the typical range of selenium level in healthy adults is typically 75–120 µg/L. Whereas our study did not quantify maternal selenium status, we determined that premature infants had altered selenium-regulated gene expression, consistent with a transcriptional profile indicative of selenium deficiency. Selenium is a trace element that plays a critical role in the synthesis and function of selenoproteins—critical constituents of the antioxidant defense system and mediators of inflammation and cellular homeostasis (Labunskyy et al. 2014). Selenoproteins play crucial roles in various fundamental cellular functions, including the reduction of hydroperoxides, the refurbishment of oxidized methionine residues, and the regulation of redox reactions in thyroid hormone metabolism (Labunskyy et al. 2014). Selenium, due to its cytoprotective properties, may serve as a therapeutic agent in numerous conditions where cellular injury is a contributing factor (Huang et al. 2012). Beyond these well-characterized roles, selenium has been further suggested to play a protective role against inappropriate or premature apoptosis, although molecular mechanisms for such a role remain unclear (Gao et al. 2019). A number of investigations have reported that selenium levels are significantly lower in both preterm infants and in mothers who deliver preterm (Gathwala et al. 2003; Gathwala and Aggarwal 2016). Furthermore, an increased incidence of preterm births has been associated with geographic regions where selenium-deficient soils are predominant (Gathwala et al. 2003). Interestingly, selenium deficiency in preterm infants has a tendency to persist beyond the neonatal period and can remain a concern of clinical significance even at four months of age (Sievers et al. 2001). Despite these observations, our review of the literature did not identify any studies specifically investigating the role of selenium deficiency in the pathogenesis of NEC in premature infants. Because NEC is a multifactorial disease with a cascade of interconnected pathologic processes, we speculate that selenium deficiency may be a significant, yet unrecognized, link in this chain (Figs. 5 and 6).

To date, relatively little research has investigated the genetic causes of NEC in preterm infants. Cuna et al. (Cuna and Sampath 2017) have conducted a comprehensive review of existing literature and speculated that the genetic alterations involved in NEC most significantly affect three primary biological pathways: inflammation, oxidative stress, and apoptosis. In another study, Chen et al. (Chen et al. 2018) analyzed gene expression profiles from intestinal tissue biopsies of NEC controls and patients. Abnormal gene expression for tryptophan metabolism and drug metabolism were indicated by their findings; however, the functional consequence and potential clinical significance of these changes remain to be elucidated. Similarly, Chan et al. (Chan et al. 2012) compared gene expression in infants with NEC and spontaneous intestinal perforation. In their study, they observed dysregulation of pro-inflammatory and anti-inflammatory genes in NEC patients and downregulation of anti-apoptotic signaling, extracellular matrix integrity, and cell adhesion genes. In line with these observations, our research also demonstrated differential gene expression of previously implicated genes in NEC pathogenesis (Fig. 5), again pointing towards the involvement of inflammation, oxidative stress, and apoptosis-related pathways in the disease process.

Jung et al. found remarkable results in transcriptome analysis in patients with NEC (Jung et al. 2017). They took tissue samples from both the sick bowel segment and the normal bowel segment of 5 preterm infants who were operated for NEC (Jung et al. 2017). They compared the two groups by analyzing transcriptome from both groups. They found that the expression of 65 genes was altered in the intestinal segments with NEC lesions. Of these genes, 57 were down-regulated and 8 were up-regulated (Jung et al. 2017). In particular, they speculated that the *DPF3* and *CAMK4* genes, whose expression is altered, may play an important role in the development of NEC (Jung et al. 2017). These two genes were down-regulated (Jung et al. 2017). In pathway analysis of genes with altered expression, they found that they were associated with thyroid cancer and axon guide pathways (Jung et al. 2017). In our study, we analyzed RNA extracted from blood samples of one healthy infant and 11 infants diagnosed with NEC. Notably, the genes identified as being differentially expressed in our cohort did not show the same expression patterns observed in previous studies. Specifically, we found that the thyroid hormone signaling pathway was significantly affected in our study samples. In contrast, Egozi et al. (Egozi et al. 2023) analyzed RNA from tissue samples obtained during surgery from 19 NEC patients and 13 control neonates. The 19 NEC patients underwent surgery due to intestinal perforation, while the 13 control neonates were operated on for spontaneous intestinal perforation. Their study focused on tissue gene expression, providing a different perspective on the molecular changes occurring in NEC. Egozi et al. (Egozi et al. 2023) found that the expression of inflammatory cytokines and chemokines was significantly increased in NEC patients by transcriptome analysis. In particular, the activation of the Toll-like receptor (TLR) signaling pathway played a significant role in their gene expression study. Observing their data, we found that certain inflammatory genes like *TNFAIP3, IL1B, IL6,* and *SERPINB1* were overexpressed in NEC patients, which was consistent with our findings. In addition, apoptosis induction in NEC patients, as reported by Egozi et al., is correlated with the apoptotic pathways we identified in our study, to further validate the role of inflammation and cell death in NEC pathogenesis (Egozi et al. 2023).

One of the most interesting findings in Egozi et al.'s (Egozi et al. 2023) study was the downregulation of SELENOP expression. Although they observed this alteration, the authors made no comment on the potential relationship between this finding and selenium deficiency. In contrast, our study found genes involved in inflammatory activation and apoptosis to be upregulated in NEC patients (Fig. 5). We also identified gene expression alterations consistent with selenium deficiency (Fig. 5), which can further contribute to NEC pathophysiology. Another noteworthy transcriptomic study on NEC is that of Xie et al. (Xie et al. 2023), who split their samples into three groups. They isolated RNA from necrotic intestinal tissue in infants with NEC, normal intestinal tissue from the same cohort, and intestinal samples from infants undergoing surgery for intestinal atresia. Two important findings were evident from their analysis of the necrotic tissue group. While the authors did not comment on the clinical significance of these observations, one of the most significant changes they reported was that of the toll-like receptor (TLR) signaling pathway (Xie et al. 2023). Consistent with this, our study also showed prominent dysregulation of the TLR signaling pathway that is closely associated with systemic inflammation in NEC (Fig. 5).

Another notable finding reported by Xie et al. (Xie et al. 2023) was the disruption of glutathione metabolism in NEC-affected tissues. Although this was mentioned among numerous other metabolic abnormalities, the authors did not mention its potential importance. Selenium is a trace element that plays a crucial role in maintaining normal glutathione metabolism, and selenium deficiency is known to suppress this pathway (Fig. 5). In our study, we observed altered gene expression typical of selenium deficiency, which we propose might be a causative agent in the development of NEC (Fig. 5). Interestingly, in all three cited studies (Jung et al. 2017; Egozi et al. 2023; Xie et al. 2023), NEC was examined primarily as a localized condition of the intestine and not with respect to its systemic nature. While those studies did recognize several significant molecular findings that are shared with our results, they did not provide an integrated hypothesis related to the role of these molecular changes in NEC etiopathogenesis.

Systemic oxidative stress with hypoxia are important steps in the emergence of NEC. In a study investigating oxidative stress in infants with NEC, premature babies were divided into 3 groups. Group 1 (NEC stage 1), Group 2 (NEC stage 2–3), Group 3 normal premature infants (Aydemir et al. 2011). They analyzed Total Oxidant Capacity (TOC) and Total Antioxidant Capacity (TAC) levels in all groups. They found that the TAC level was similar in the 3 groups, but the TOC level was higher in Group 2 (Aydemir et al. 2011). The most important components that make up TOC are reactive oxygen species formed as a result of hypoxia. Reactive oxygen species are critical in the emergence of NEC. Reactive oxygen species are formed due to hypoxia and when they rise above the critical level in the cell, autophagy and apoptosis are activated. In their experimental study, Tian et al. examined the expression of autophagy genes in mice with NEC (Tian et al. 2022). Autophagy is a process that protects the cell from stressful situations. If autophagy is activated unnecessarily, it causes cell death. Hypoxia, oxidative stress and endoplasmic reticulum stress are the main causes of activation autophagy unnecessarily. In a study, it was suggested that the genes *HIF-1 A, ITGA3, VEGFA* and *ITGB4*, known as autophagy genes, were altered in mice with NEC (Tian et al. 2022). As a result of our study, we found that gene expression of these autophagy changing in NEC patients, indicating that autophagy is activated (Fig. 5). Based on our findings, we observed upregulation of genes associated with hypoxic signaling, suggesting that hypoxia may serve as a critical initiating event in the cascade leading to NEC. We propose that hypoxia-induced oxidative stress acts as a key driver of systemic inflammation, contributing significantly to the pathogenesis of NEC in preterm infants (Fig. 6). These results support the hypothesis that oxidative stress plays a central role in the development of NEC.

Undoubtedly, the most important link in the chain in the emergence of NEC is increased apoptosis (Kim and Albenese 2006). In the literature, the relationship between NEC and apoptosis has been examined and it has been shown that the earliest onset finding is apoptosis in intestinal villus crypts. Claims and studies on this subject have examined the phenomenon at the scale of gastrointestinal tract cells (Dotinga et al. 2023; Yu et al. 2024; Leaphart et al. 2007). We found signs of systemically occurring apoptosis in all NEC-presenting infants in our study. We speculate that apoptosis may have been activated not only in the villus crypts, but in fact in every system in the body of these babies. Our study revealed a significant upregulation of apoptosis-related genes, indicating activation of the apoptotic pathway in infants with NEC (Fig. 5). We propose that this widespread apoptotic activity is driven by systemic hypoxia, oxidative stress, and subsequent activation of systemic inflammation. Under such conditions, localized gastrointestinal interventions such as the use of breast milk, while undeniably beneficial may not be sufficient on their own to fully address the underlying pathophysiological processes. Although the protective effects of breast milk are well-established and beyond the scope of this discussion, our findings suggest that additional therapeutic strategies targeting systemic oxidative stress may be necessary to interrupt this pathological cycle and improve outcomes in premature infants at risk of NEC. There are some restrictions on this study. With gene expression analyses performed on 11 NEC patients compared to just one control subject, the sample size was small. Furthermore, there is no supporting evidence from tissue-specific analyses or protein expression; all conclusions are based solely on gene expression data derived from peripheral blood samples.

## Conclusion

In this study, global gene expression profiling in infants with necrotizing enterocolitis (NEC) revealed significant dysregulation of 1,204 genes compared to healthy controls, with major alterations observed in pathways related to hypoxia, oxidative stress, inflammation, and apoptosis. Furthermore, gene expression patterns suggested a potential link between prematurity, selenium deficiency, and oxidative stress. These findings support the hypothesis that maternal selenium deficiency and oxidative stress may contribute to preterm birth, thereby increasing the risk of NEC development in neonates. Accordingly, interventions aimed at optimizing maternal antioxidant status and selenium sufficiency during pregnancy could play a preventive role in reducing the incidence of preterm birth. Additionally, postnatal strategies to ensure adequate oxygenation and selenium levels in premature infants may mitigate NEC risk. Collectively, these insights highlight two critical windows for NEC prevention: during pregnancy and immediately after birth. Finally, given selenium’s role in modulating oxidative damage, future research into its therapeutic potential may extend to other diseases characterized by oxidative stress and tissue injury**.**

## Supplementary Information

Below is the link to the electronic supplementary material.

Supplementary file1 Heat map of Top 20 DEGs (JPG 75 KB)

Supplementary file2 Graph Level 2 Pie Chart of #Seqs [Biological Process] (JPG 37 KB)

Supplementary file3 Graph Level 2 Pie Chart of#Seqs [Molecular Function] (JPG 30 KB)

Supplementary file4 (DOCX 13 KB)

Supplementary file5 (DOCX 18.6 KB)

Supplementary file6 (DOCX 20 KB)

Supplementary file7 (XLSX 95 KB)

Supplementary file8 (DOCX 15 KB)

Supplementary file9 (XLSX 648 KB)

Supplementary file10 (XLSX 34 KB)

Supplementary file11 (XLSX 31 KB)

## Data Availability

The RNAseq datasets were uploaded to the NCBI SRA platform, Accession Code: SUB15264887.
