# Supplementary figures and images for: Selenium deficiency is functionally linked with the molecular etiopathogenesis of necrotizing enterocolitis (NEC)
Source: Funct Integr Genomics. 2025 Jun 3;25(1):118. doi: 10.1007/s10142-025-01628-8 (PMC12134042; doi:10.1007/s10142-025-01628-8)

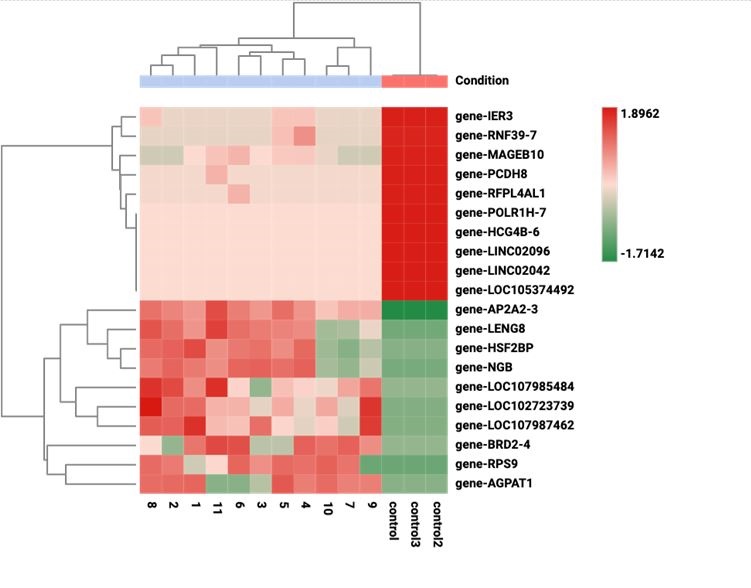

Supplement: Supplementary file 1 — Supplementary file1 Heat map of Top 20 DEGs (JPG 75 KB) [file 10142_2025_1628_MOESM1_ESM.jpg]

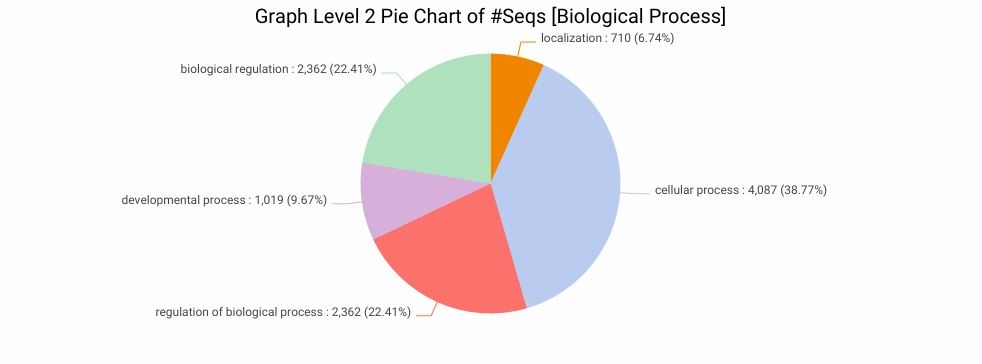

Supplement: Supplementary file 2 — Supplementary file2 Graph Level 2 Pie Chart of #Seqs [Biological Process] (JPG 37 KB) [file 10142_2025_1628_MOESM2_ESM.jpg]

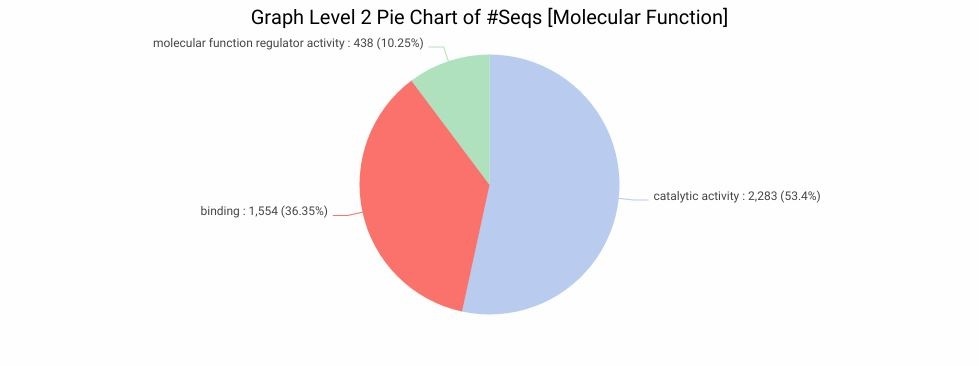

Supplement: Supplementary file 3 — Supplementary file3 Graph Level 2 Pie Chart of#Seqs [Molecular Function] (JPG 30 KB) [file 10142_2025_1628_MOESM3_ESM.jpg]
