# Supplementary material for: Selenium deficiency is functionally linked with the molecular etiopathogenesis of necrotizing enterocolitis (NEC)
Source: Funct Integr Genomics. 2025 Jun 3;25(1):118. doi: 10.1007/s10142-025-01628-8 (PMC12134042; doi:10.1007/s10142-025-01628-8)
Supplement: Supplementary file 4 — Supplementary file4 (DOCX 13 KB) [file 10142_2025_1628_MOESM4_ESM.docx]

| **Supplemetary Table 1.** Necrotizing Enterocolitis Staging System From Bell | |
| --- | --- |
| **Stage 1 (Suspected)** |  |
|  | Any one or more historical factors producing perinatal stress. |
|  | Systemic manifestations- temperature instability, lethargy, apnea bradycardia. |
|  | Gastrointestinal manifestations- poor feeding, increasing pregavage residuals, emesis (may be bilious or test positive for occult blood), mild abdominal distention, occult blood in stool (no fissure). |
|  | Abdominal radiographs showing distention with mild ileus. |
| **Stage 2 (Definite)** |  |
|  | Any one or more historical factors. |
|  | Above signs and symptoms plus persistent occult or gross gastrointestinal bleeding, marked abdominal distention. |
|  | Abdominal radiographs showing significant intestinal distention with ileus, small bowel seperation (edema in bowel wall or peritoneal fluid), unchanging or persistent "rigid" bowell loops, pneumotosis intestinalis, portal venous gas. |
| **Stage 3 (Advanced)** |  |
|  | Any or more historical factors. |
|  | Above signs and symptoms plus deterioration of vital signs, evidence of septic shock, or marked gastrointestinal hemorrhage. |
|  | Abdominal radio graphs showing pneumoperitoneum in addition to the findings listed for stage II. |
