# Supplementary material for: Selenium deficiency is functionally linked with the molecular etiopathogenesis of necrotizing enterocolitis (NEC)
Source: Funct Integr Genomics. 2025 Jun 3;25(1):118. doi: 10.1007/s10142-025-01628-8 (PMC12134042; doi:10.1007/s10142-025-01628-8)
Supplement: Supplementary file 5 — Supplementary file5 (DOCX 18.6 KB) [file 10142_2025_1628_MOESM5_ESM.docx]

| **Supplementary Table 2.** Demographic information of the patients | | | | | | |  |  |  |
| --- | --- | --- | --- | --- | --- | --- | --- | --- | --- |
|  | **Gender** | **Birth weight** | **Birth week** | **Day of surgery** | **Operation** | **Stage in Bell classification** | **Prognosis** | **Occult blood in stool** | **Pneumoperitoneum** |
| **Patient 1** | M | 765gr | 24w | 16 | Resection + ileostomy | Stage 3 | He is alive | Positive | Positive |
| **Patient 2** | M | 800gr | 25w | 14 | Resection + ileostomy | Stage 3 | He is alive | Positive | Positive |
| **Patient 3** | F | 1010gr | 29w | 13 | Resection + ileostomy | Stage 3 | She is alive | Positive | Positive |
| **Patient 4** | M | 920gr | 26w | 9 | Resection + ileostomy | Stage 3 | He is alive | Positive | Positive |
| **Patient 5** | F | 1050gr | 29w | 13 | Resection + ileostomy | Stage 3 | She is alive | Positive | Positive |
| **Patient 6** | M | 1130gr | 31w | 14 | Resection + ileostomy | Stage 3 | He is alive | Positive | Positive |
| **Patient 7** | F | 1080gr | 29w | 11 | Resection + ileostomy | Stage 3 | She is alive | Positive | Positive |
| **Patient 8** | F | 1000gr | 28w | 14 | Resection + ileostomy | Stage 3 | She died | Positive | Positive |
| **Patient 9** | M | 785gr | 24w | 11 | Resection + ileostomy | Stage 3 | He is alive | Positive | Positive |
| **Patient 10** | F | 900gr | 26w | 10 | Resection + ileostomy | Stage 3 | She died | Positive | Positive |
| **Patient 11** | F | 750gr | 24w | 11 | Resection + ileostomy | Stage 3 | She is alive | Positive | Positive |
| **Control** | F | 3560gr | 40W |  |  |  | She is alive | |  |
