# Supplementary material for: Selenium deficiency is functionally linked with the molecular etiopathogenesis of necrotizing enterocolitis (NEC)
Source: Funct Integr Genomics. 2025 Jun 3;25(1):118. doi: 10.1007/s10142-025-01628-8 (PMC12134042; doi:10.1007/s10142-025-01628-8)
Supplement: Supplementary file 6 — Supplementary file6 (DOCX 20 KB) [file 10142_2025_1628_MOESM6_ESM.docx]

**Supplementary Table 3.** Sequencing statistics and Quality Control (QC) information for samples

| Sample | Test | Raw  reads | Clean  reads | Raw  Base  (G) | Clean Base  (G) | Effective Rate  (%) | Error  Rate  (%) | Q20  (%) | Q30  (%) | GC Content (%) |
| --- | --- | --- | --- | --- | --- | --- | --- | --- | --- | --- |
| *Control* | *Control* group* | *37,449,888* | *36,273,858* | *5.62* | *5.44* | *96.86* | *0.03* | *96.88* | *91.75* | *51.77* |
| Patient 1 | Test group | 49,824,192 | 488,249,24 | 7.47 | 7.32 | 97.99 | 0.01 | 98.58 | 96.08 | 52.14 |
| Patient 2 | Test group | 51,970,064 | 51,012,122 | 7.8 | 7.65 | 98.16 | 0.01 | 98.54 | 96.01 | 52.04 |
| Patient 3 | Test group | 49,629,482 | 48,792,238 | 7.44 | 7.32 | 98.31 | 0.01 | 98.56 | 96.1 | 52.08 |
| Patient 4 | Test group | 43,982,176 | 43,216,758 | 6.6 | 6.48 | 98.26 | 0.01 | 98.52 | 95.97 | 51.89 |
| Patient 5 | Test group | 46,328,822 | 45,547,162 | 6.95 | 6.83 | 98.31 | 0.01 | 98.58 | 96.13 | 51.17 |
| Patient 6 | Test group | 48,109,192 | 47,215,812 | 7.22 | 7.08 | 98.14 | 0.01 | 98.53 | 95.96 | 52.51 |
| Patient 7 | Test group | 39,469,194 | 38,426,626 | 5.92 | 5.76 | 97.36 | 0.01 | 98.45 | 95.75 | 51.54 |
| Patient 8 | Test group | 43,084,738 | 42,095,762 | 6.46 | 6.31 | 97.70 | 0.01 | 98.43 | 95.74 | 51.51 |
| Patient 9 | Test group | 40,043,064 | 39,074,218 | 6.01 | 5.86 | 97.58 | 0.01 | 98.74 | 96.57 | 51.66 |
| Patient 10 | Test group | 45,947,742 | 44,978,280 | 6.89 | 6.75 | 97.89 | 0.01 | 98.72 | 96.52 | 51.73 |
| Patient 11 | Test group | 53,570,550 | 51,091,768 | 8.04 | 7.66 | 95.37 | 0.01 | 98.45 | 95.97 | 50.86 |

*the healthy infant control dataset was obtained from our previous study Gürünlüoğlu K et al. Funct Integr Genomics. 2022; 22: 359-369.
