# Supplementary material for: Selenium deficiency is functionally linked with the molecular etiopathogenesis of necrotizing enterocolitis (NEC)
Source: Funct Integr Genomics. 2025 Jun 3;25(1):118. doi: 10.1007/s10142-025-01628-8 (PMC12134042; doi:10.1007/s10142-025-01628-8)
Supplement: Supplementary file 8 — Supplementary file8 (DOCX 15 KB) [file 10142_2025_1628_MOESM8_ESM.docx]

## Supplementary Table 5. The DEGs analysis statistics

| ComparisonGroups | TotalDEGs number(Probability > 0.9) | Up-regulatedgene number(M>0) | Down-regulated gene number(M<0) |
| --- | --- | --- | --- |
| patient-1 vs control | 2,975 | 1,607 | 1,368 |
| patient-2 vs control | 1,818 | 972 | 846 |
| patient-3 vs control | 1,139 | 606 | 533 |
| patient-4 vs control | 1,523 | 782 | 741 |
| patient-5 vs control | 1,432 | 725 | 707 |
| patient-6 vs control | 1,662 | 816 | 846 |
| patient-7 vs control | 2,540 | 1,232 | 1,308 |
| patient-8 vs control | 3,029 | 1,512 | 1,517 |
| patient-9 vs control | 3,678 | 1,854 | 1,824 |
| patient-10 vs control | 2,960 | 1,624 | 1,336 |
| patient-11 vs control | 2,955 | 1,467 | 1,479 |
| ComparisonGroups | TotalDEGs number(FDR < 0.05) | Up-regulatedgene number(logFC > 1) | Down-regulated gene number(logFC < -1) |
| all-patients vs control | 1,204 | 636 | 568 |
